# Endoscopic submucosal dissection for removal of a large hypopharyngeal lipoma with oval forceps traction

**DOI:** 10.1055/a-2587-9335

**Published:** 2025-05-09

**Authors:** Tian-Xing Yuan, Ye-Han Zhou, Yu Bao, Rui Zhao

**Affiliations:** 1 12599 School of Medicine, University of Electronic Science and Technology of China, Chengdu, China; 2 92293 Department of Pathology, Sichuan Cancer Hospital and Institute, Chengdu, China; 3 92293 Department of Endoscopy, Sichuan Cancer Hospital and Institute, Chengdu, China

A 59-year-old man presented with a mass in the hypopharynx found during routine screening. Endoscopic examination revealed a large submucosal swelling in the posterior hypopharynx and cervical esophagus, with the base located in the posterior hypopharyngeal wall ( Fig. 1 ). Contrast-enhanced CT showed a mass with fat density, which did not enhance ( Fig. 2 ), consistent with a lipoma.

**Fig. 1:**
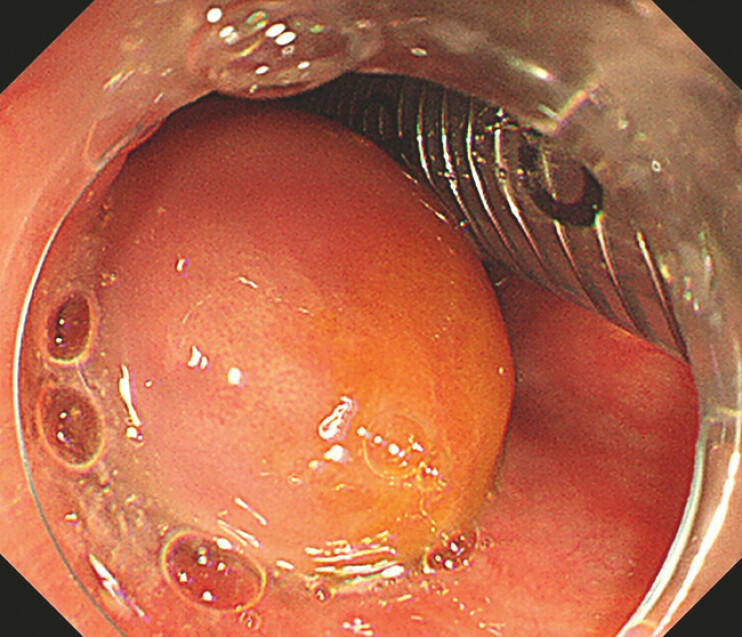
Endoscopic findings. The base located in the posterior hypopharyngeal wall.

**Fig. 2:**
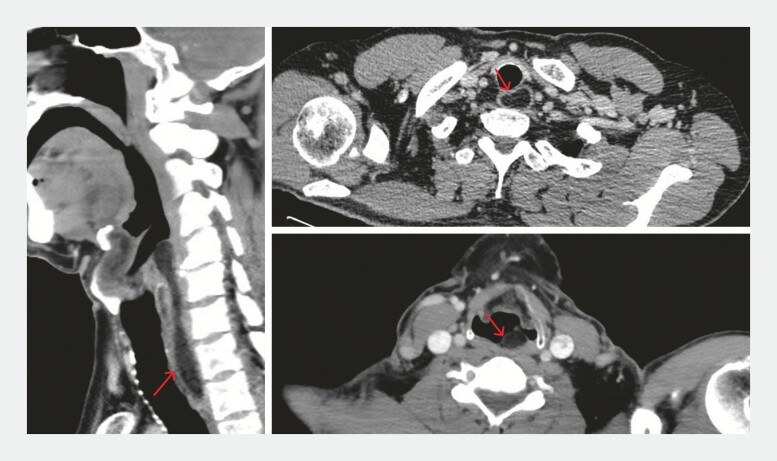
Contrast-enhanced CT showed a mass with fat density, without significant contrast enhancement.

The tumor was successfully removed ( Fig. 3 ) using endoscopic submucosal dissection (ESD) assisted by oval forceps ( Video 1 ). The oval forceps provided optimal traction, ensuring clear exposure of the broad tumor base, which facilitated its complete removal. Due to the broad base of the tumor, snare traction was relatively difficult to perform. Oval forceps, however, provided simpler operation and allowed for better visualization of the tumor base. The procedure took 57 minutes, with no major complications during or after the surgery.

**Fig. 3:**
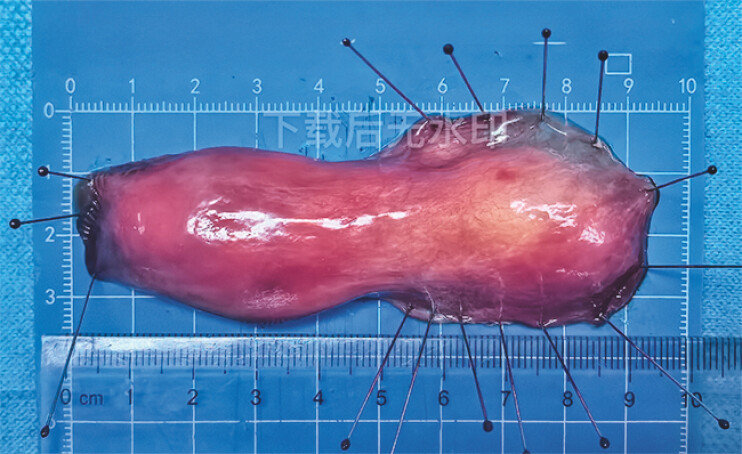
The specimen measured 9.5 cm in length and 3.5 cm in width at its widest point.

Endoscopic submucosal dissection was performed to remove a large hypopharyngeal lipoma with oval forceps traction.Video 1

Histopathological analysis confirmed the diagnosis of submucosal lipoma. Fluorescence in situ hybridization (FISH) testing revealed no MDM2 gene amplification, excluding liposarcoma ( Fig. 4 ).

**Fig. 4:**
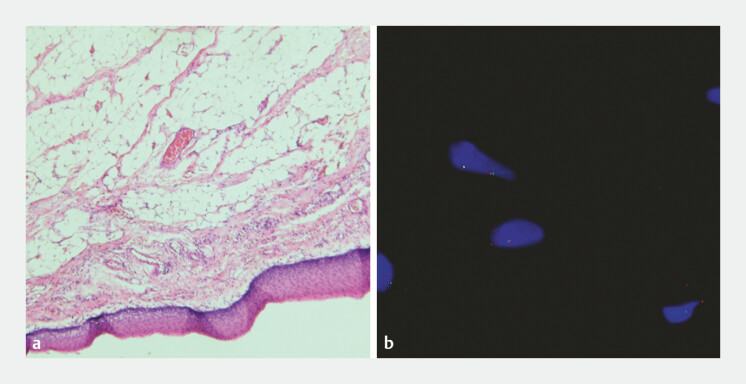
Histological analysis revealed a submucosal lipoma. **a** Hematoxylin and eosin-stained images (×50). **b** FISH testing revealed no MDM2 gene amplification. Abbreviation: FISH, fluorescence in situ hybridization.

Lipomas are common benign tumors but are rare in the hypopharynx 1 . To our knowledge, there have been reports using snare traction-assisted ESD to remove hypopharyngeal tumors 2 , but the use of oval forceps for ESD in large hypopharyngeal lipomas has not been previously described.

This case highlights the feasibility, safety, and effectiveness of oval forceps-assisted ESD in treating large hypopharyngeal lipomas, particularly those with a broad base. This method offers a practical, minimally invasive option for the endoscopic management of these rare tumors.

Endoscopy_UCTN_Code_CCL_1AB_2AB

Endoscopy_UCTN_Code_TTT_1AO_2AG_3AD

## Notes

Endoscopy E-Videos https://eref.thieme.de/e-videos
*E-Videos* is an open access online section of the journal *Endoscopy* , reporting on interesting cases and new techniques in gastroenterological endoscopy. All papers include a high-quality video and are published with a Creative Commons CC-BY license. Endoscopy E-Videos qualify for HINARI discounts and waivers and eligibility is automatically checked during the submission process. We grant 100% waivers to articles whose corresponding authors are based in Group A countries and 50% waivers to those who are based in Group B countries as classified by Research4Life (see: https://www.research4life.org/access/eligibility/ ). This section has its own submission website at https://mc.manuscriptcentral.com/e-videos .
